# A review on bacterial resistance to carbapenems: epidemiology, detection and treatment options

**DOI:** 10.2144/fsoa-2019-0098

**Published:** 2020-01-27

**Authors:** Ann A Elshamy, Khaled M Aboshanab

**Affiliations:** 1 Department of Microbiology & Immunology, Faculty of Pharmacy, Ain Shams University, POB 11566, Cairo, Egypt

**Keywords:** antimicrobial resistance, carbapenem, carbapenem resistance, carbapenem-producing *Enterobacteriaceae*, carbapenem-resistant *Enterobacteriaceae*, carbapenemase, MDR, multidrug-resistance

## Abstract

Carbapenems are a class of antimicrobial agents reserved for infections caused by multidrug-resistant microorganisms. The emergence of carbapenem resistance has become a serious public health threat. This type of antimicrobial resistance is spreading at an alarming rate, resulting in major outbreaks and treatment failure of community-acquired and nosocomial infections caused by the clinically relevant carbapenem-producing *Enterobacteriaceae* or carbapenem-resistant *Enterobacteriaceae*. This review is focused on carbapenem resistance, including mechanisms of resistance, history and epidemiology, phenotypic and genotypic detection in the clinically relevant bacterial pathogens and the possible treatment options available.

## Lay abstract

Carbapenems are antimicrobial drugs used for treating infections caused by bacteria that are resistant to multiple antibiotics. In recent years, the rise of carbapenem resistance has become a serious public health threat. Carbapenem resistance is spreading rapidly, causing several outbreaks and treatment failure of many infections. This review focuses on bacterial resistance to carbapenems, including mechanisms of resistance, the history and spread of such resistance, how to detect carbapenem resistance in bacteria causing infections. It also discusses the possible options for treatment.

## The emergence & spread of antimicrobial resistance

The risk of antimicrobial resistance (AMR) is rapidly increasing worldwide [1]. Governments all over the globe are starting to pay attention to such a serious threat to modern medicine. The emergence of AMR is a natural phenomenon in microorganisms, yet it is augmented by the overuse of antimicrobial agents in both humans and animals [2,3]. Although antimicrobials are among the most commonly used agents in modern medicine, approximately 50% of prescribed antimicrobials are considered unnecessary. This overuse of antimicrobials is a major driving force toward AMR [4]. The scarcity of new antimicrobials to replace those that have become ineffective necessitates our need to protect the effectiveness of existing agents [2,3]. Some bacteria are intrinsically resistant to more than one class of antimicrobial agents. Cases of acquired resistance are of greater concern; where previously susceptible bacteria acquire resistance to an antimicrobial agent under the selective pressure of use of such agent. Resistance that develops due to chromosomal mutation is termed vertical evolution, while that gained through the acquisition of genetic material from other resistant organisms is termed horizontal evolution [5].

A major reason for the rapid spread of AMR through bacterial populations is that genes conferring resistance are carried on plasmids or on other highly movable genetic elements that are independently replicated and passed between bacterial cells and species. Once a newly discovered antimicrobial agent is proven to be effective and is approved for therapeutic use, clinically significant resistance often appears in months to years [6].

The two most commonly used systems for antimicrobial susceptibility testing worldwide are the Clinical and Laboratory Standards Institute (CLSI) and the European Committee for Antimicrobial Susceptibility Testing [3].

## The discovery of carbapenems

**Figure 1.**
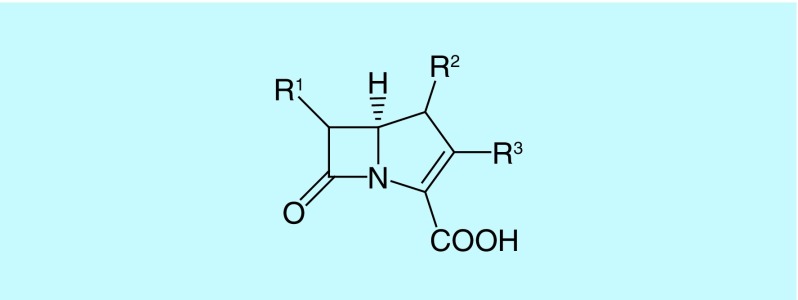
Carbapenem backbone structure.

Carbapenems are β-lactam antibiotics possessing a β-lactam ring and a five-membered ring which differs from that of penicillin in being unsaturated and having a carbon atom rather than sulfur (Figure 1) [7,8].

**Figure 2.**
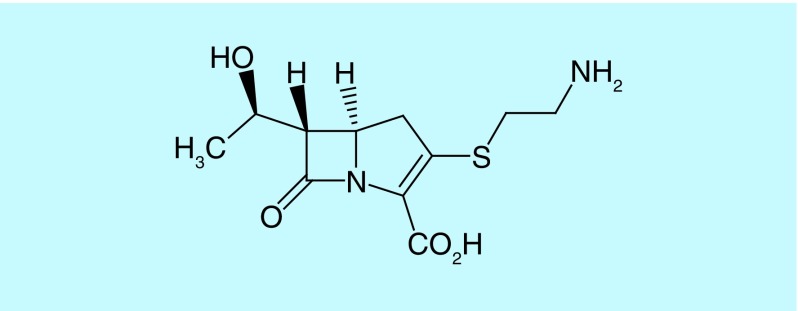
The chemical structure of thienamycin.

This unique molecular structure confers remarkable stability against the majority of β-lactamases, including extended spectrum β-lactamases (ESBLs) [9,10]. In 1976, thienamycin, a naturally derived product of *Streptomyces cattleya*, was the first discovered carbapenem (Figure 2) [11,12].

**Figure 3.**
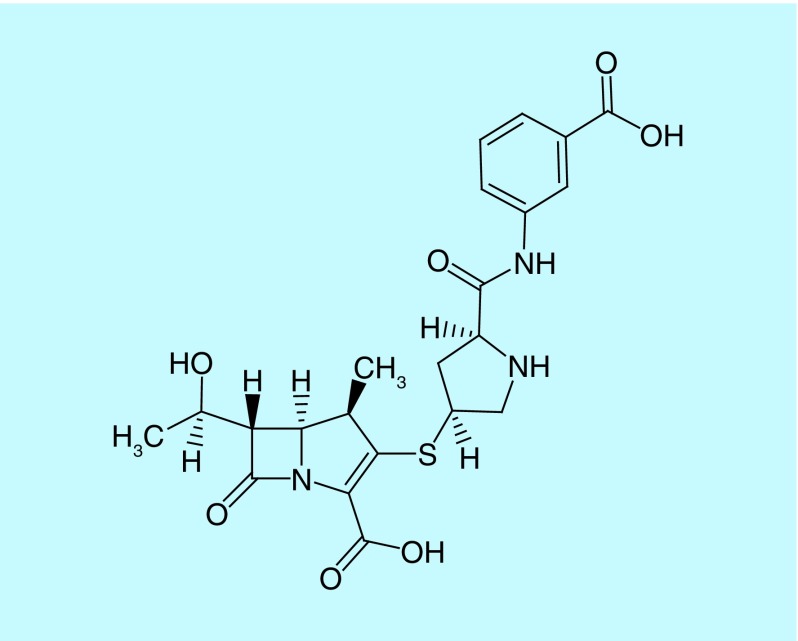
Chemical structure of imipenem.

Thienamycin's instability in water limited its clinical use [13]. However, this instability was overcome by the semisynthetic production of its N-formimidoyl derivative, called imipenem (Figure 3) [14,15]. Imipenem is degraded by a renal tubular dipeptidase enzyme, dehydropeptidase I. For this reason, imipenem is co-administered with cilastatin, a competitive antagonist, which inhibits imipenem’s renal degradation [16]. Cilastatin also protects the kidneys from the toxic effects caused by higher doses of imipenem [15–17].

## Currently marketed carbapenems, their spectrum of activity & indications

**Figure 4.**
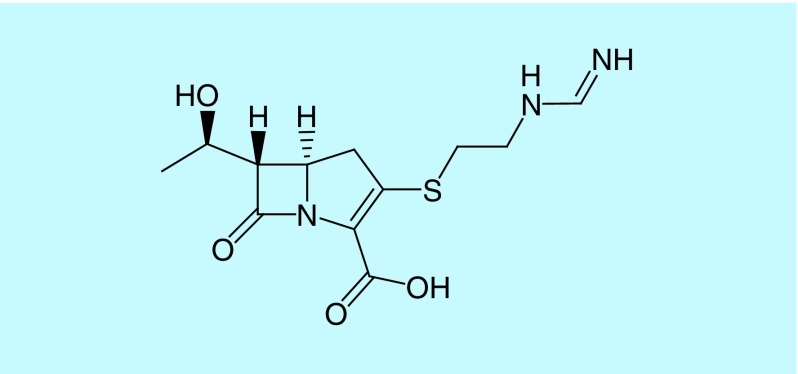
Chemical structure of meropenem.

Carbapenems such as imipenem/cilastatin, meropenem, doripenem and ertapenem are the latest developed β-lactams currently available in the market possessing a broad spectrum of activity and are usually reserved for treating infections caused by multidrug-resistant (MDR) pathogens [13,18–20]. Imipenem/cilastatin is used for the treatment of a wide variety of infections, including urinary tract infections and lower respiratory tract infections, especially in cases of infections caused by cephalosporin-resistant bacteria. Meropenem does not need to be administered with cilastatin, as it is not sensitive to the dehydropeptidase I enzyme. Compared with imipenem, meropenem is less active against Gram-positive bacteria (especially *Enterococcus*) and more active against Gram-negative bacteria. The pyrrolidinyl substituent at the 2-position of meropenem’s side chain (Figure 4) is thought to be responsible for the improved activity against Gram-negative bacteria and stability toward the dehydropeptidase I enzyme [17,21].

**Figure 5.**
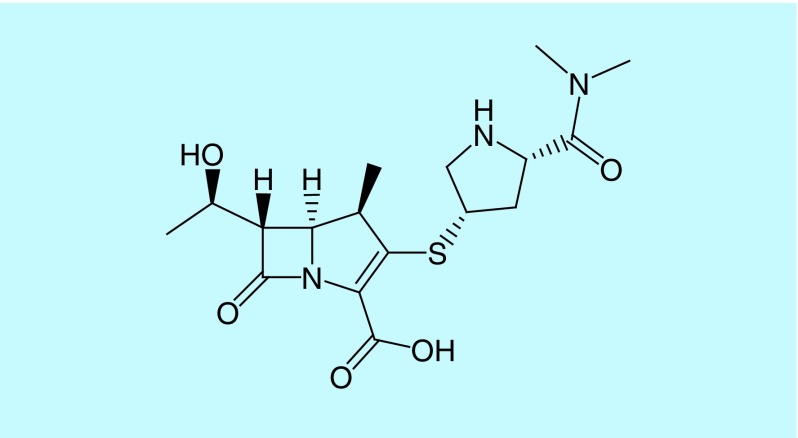
Chemical structure of ertapenem.

Doripenem's spectrum of activity is similar to that of meropenem, with better activity against some resistant *Pseudomonas* strains. Ertapenem (Figure 5) has lower activity against *Pseudomonas aeruginosa*, *Enterococcus* and species of *Acinetobacter* than imipenem and meropenem but has a longer half-life which allows once-daily dosing. Ertapenem shows good activity against *Enterobacteriaceae* and anaerobes [7] and is considered one of the first-line treatment options for the empiric treatment of community-acquired intra-abdominal infections, as recommended by the Infectious Disease Society of America (VA, USA) [22, 23]. Doripenem, imipenem and meropenem are recommended for high-risk nosocomial and community-acquired abdominal infections [22].

## Mechanism of action of carbapenems

Bacterial cell walls are complex structures composed of a peptidoglycan polymer. The last transpeptidation step in the synthesis of peptidoglycan is enabled by transpeptidase enzymes, which are penicillin-binding proteins (PBPs). The structure of carbapenems (and other β-lactams) is closely related to acylated D-alanyl-D-alanine – the terminal amino acid residues of the peptidoglycan. This structural similarity allows carbapenems to bind irreversibly to the active site of the PBPs, leading to the inhibition of transpeptidation of the peptidoglycan layer via crosslinking, this in turn disrupts the cell wall synthesis [24,25]. At last, bacterial cell death results from the continued activity of autolysins, a group of bacterial surface enzymes. It is speculated that the biological role of autolysins is to create nicks in the cell wall that function as attachment points for new peptidoglycan units. Thus, inhibition of cell wall biosynthesis by β-lactam agents, in association with continued cell wall autolysis, creates weak spots in the cell wall through which the cell membrane extrudes. Since the cell membrane is not strong enough to keep the hypertonic cell from rupturing by osmotic shock, it eventually ruptures [26,27].

## Adverse reactions to carbapenems

The most common adverse reactions to carbapenems are nausea and vomiting, occurring in approximately 1–20% of treated patients. Seizures have been reported in 1.5% of patients, particularly with high doses. Patients with allergies to other β-lactams may experience hypersensitivity reactions, although the incidence of immediate hypersensitivity is low (<1%) [7].

## The emergence of carbapenem resistance

Since 2000, the number of bacterial species carrying *ESBL* genes has increased, and community-acquired *Escherichia coli* isolates with the ability to produce ESBLs that hydrolyze almost all β-lactam agents, except for carbapenems, have been reported worldwide [28,29]. As a result, the clinical use of carbapenems has increased. This in turn caused an increase in the number of clinical bacterial isolates producing β-lactamases that have the ability to hydrolyze carbapenems, known as carbapenemases [30]. Thus, the overuse of carbapenems has led to the emergence of carbapenem resistance, which is the ability of bacteria to grow and survive in the presence of clinically relevant carbapenem concentrations [31].

## Mechanism of carbapenem resistance

Resistance to carbapenems may be attributed to three major mechanisms: porin-mediated resistance to reduce uptake of carbapenems, efflux pumps, which pump the carbapenem outside the cells and enzyme-mediated resistance which is mediated via the acquisition of carbapenemase genes. The reduced uptake or increased efflux of antibiotics are usually associated with an overexpression of β-lactamases possessing weak affinities for carbapenems [32,33]. The nature of the resistance determinants can affect the dynamics of their spread [34].

### Porin-mediated resistance

Bacteria can limit the entry of carbapenems into the periplasmic space where PBPs are located. This mechanism involves the modification of porin expression or alterations in the porin-encoding gene, leading to either complete loss or defects in the respective porin [35]. For example, the main mechanism of resistance to carbapenems in *P. aeruginosa* isolates is the downregulation of the gene encoding the orpd porin [36]. Likewise, the altered expression of ompk35 and ompk36 in *Klebsiella pneumoniae* was observed to cause a high resistance level to ertapenem [37].

### Overproduction of efflux pumps

Efflux pumps are generally able to recognize numerous substrates, given that affinity is based on physiochemical properties (e.g., electric charge, aromatic or hydrophobic properties) instead of chemical structures. This explains the presence of MDR efflux pumps which can expel many structurally unrelated antimicrobials [38]. Gram-negative bacteria such as *P. aeruginosa* and *Acinetobacter* species are well known for their efflux-mediated β-lactam resistance [39]. The overexpression of efflux pumps active on carbapenems may lead to carbapenem resistance [10,40].

### Enzyme-mediated resistance

In most cases, resistance is due to the production of β-lactamases capable of hydrolyzing carbapenems and other β-lactam antimicrobials, hence they are called carbapenemases. This resistance mechanism poses the greatest threat, as these enzymes can inactivate the majority of β-lactams and are encoded by genes carried on transposons, plasmids or other mobile genetic elements, which can be horizontally transferred to other bacterial species [10].

Based on their molecular structures, carbapenemases belong to three classes of β-lactamases; class A, B and D. Classes A and D possess a serine residue at the active site to facilitate ring opening, they are thus called serine β-lactamases (SBLs) [41]. Class B comprises metallo-β-lactamases (MBLs), the active site of which uses zinc ions to mediate bond hydrolysis [39]. β-lactamase inhibitors such as clavulanic acid, sulbactam and/or tazobactam can inhibit SBLs. On the other hand, MBLs are not affected by such inhibitors, but are inhibited by metal ion chelators, such as dipicolinic acid, EDTA or o-phenanthroline; all of which are not approved for clinical use [42].

### Class A carbapenemases

Class A carbapenemases include *K. pneumoniae* carbapenemases (KPCs), imipenem-hydrolyzing β-lactamase (IMI), Guiana extended spectrum carbapenemase (GES), *Serratia fonticola* carbapenemase, *Serratia marcescens* enzyme and nonmetallo-carbapenemase-A [43]. KPCs have the ability to hydrolyze all β-lactams and strains carrying the *bla_KPC_* gene are usually resistant to other antimicrobials, such as aminoglycosides, fluoroquinolones and trimethoprim-sulfamethoxazole, making them MDR. Thirteen KPC variants have been described so far [44]. The most frequently reported of which are *KPC-2* and *KPC-3* [45,46]. The *bla_KPC_* genes are plasmid-encoded, and are thus prone to interspecies horizontal transmission [47].

Isolates that produce IMI might rarely be detected due to their unusual AMR profile, such isolates are usually resistant to imipenem, but show intermediate resistance to ertapenem and sensitive toward extended-spectrum cephalosporins. Moreover, the *bla_IMI_* gene is not included in the panel of genes targeted by commercially available molecular diagnostic kits. IMI-1 carbapenemases are chromosomally encoded and are thus considered clinically irrelevant [48].

Several genotypes of the *bla_GES_* gene (coding for GES β-lactamase) contain a point mutation (*G493A*), which causes the incorporation of serine instead of glycine. The resulting mutant enzyme displays carbapenemase activity. Reports of GES carbapenemases are rare but increasing steadily [49]. As the case of KPC, GES carbapenemases are plasmid-borne [28,50].

### Class B carbapenemases

In 1966, Sabath and Abraham discovered the first class B enzyme BCII, the *Bacillus cereus* MBL [51]. By 1989, only four MBLs were discovered, and were all chromosomally encoded, consequently they were deemed clinically unimportant. Yet, in 1991, the plasmid-encoded imipenem-resistant *Pseudomonas*-type carbapenemases (IMP) was discovered in *P. aeruginosa* in Japan, which revived the clinical interest in this class of enzymes [52]. Today, MBLs are mainly plasmid-encoded, facilitating their transmission among microbial pathogens [53]. They are also the most molecularly diverse class of carbapenemases and can inactivate the majority of β-lactams, with the exception of monobactams [54]. New Delhi MBL (NDM) is an MBL that can confer resistance to enteric pathogens, such as *K. pneumoniae* and *E. coli*, making them resistant to β-lactams, including carbapenems [55] but not aztreonam [56]. Verona integron-encoded MBL (VIM) was first described in Verona, Italy, from a *P. aeruginosa* isolate in 1999. The hydrolytic profile of VIM is like other members of this class, hydrolyzing most β-lactams except for aztreonam [53]. It is worth mentioning that bacteria co-expressing SBLs and MBLs are usually able to hydrolyze the clinically relevant monobactam, aztreonam [57]. Moreover, two MBLs, including German imipenemase and Sao Paulo MBL have been detected in the clinical isolates of *S. marcescens* and *P. aeruginosa*, respectively [58,59].

### Class D carbapenemases

These include the oxacillinase (OXA) enzymes, which have the ability to efficiently hydrolyze oxacillin, for which they were named [60]. The OXA-2 β-lactamase was the first discovered class D enzyme [61]. The carbapenem-hydrolyzing OXA-48 enzyme has high hydrolysis activity toward penicillins and low hydrolysis activity toward carbapenems [33]. It is also not affected by β-lactamase inhibitors, which is why this enzyme has recently gained attention [62]. Other OXA β-lactamases as OXA-23, OXA-24/40 and OXA-58, are frequently found in species of *Acinetobacter* but have a relatively weak carbapenemase activity [30]. One of the greatest threats posed by this class of enzymes is the lack of inhibitors for them [60].

## History & epidemiology of the most clinically encountered carbapenemases

### *Klebsiella pneumoniae* carbapenemases

In 1996, the first KPC enzyme (KPC-2) was isolated and characterized in North Carolina, USA, from a *K. pneumoniae* clinical isolate [63,64]. Since then, KPC-producing *K. pneumoniae* isolates have widely disseminated across the US [65]. KPC-producers’ outbreaks have since been reported worldwide. In 2007, a hospital in Crete, Greece, reported an outbreak caused by KPC-2-producing *K. pneumoniae* isolates. The outbreak affected 22 hospitalized patients who had no history of travelling to KPC-producer infested areas [66]. The first KPC identified in *P. aeruginosa* (outside the *Enterobacteriaceae* family) was in Medellin, Colombia [67]. In 2008, an outbreak of KPC-3-producing *K. pneumoniae* was reported in Columbia, causing the death of 20 out of 32 (62.5%) affected patients. In 2009, another outbreak was reported in Italy, also caused by a KPC-3-producing *K. pneumoniae* isolate, which affected 16 intensive care unit patients [68]. In 2010, KPC-2-producing *Citrobacter freundii* isolates were reported in Madrid, Spain [69]. A study on carbapenem-resistant *Enterobacteriaceae* (CRE) isolates obtained during the period between 2013 and 2016 at a health system in Northern California reported that 38.7% of the tested isolates harbored carbapenemase genes, 20.8% of which carried the *bla_KPC_* gene [70].

### New Delhi MBL

In 2008, an NDM-producing *K. pneumoniae* isolate was identified in a Swedish patient of Indian origin who had recently been to New Delhi, India, where he acquired a urinary tract infection caused by a carbapenem-resistant *K. pneumoniae* isolate [55]. Later on, the SENTRY Antimicrobial Surveillance Program reported that NDM-producing *Enterobacteriaceae* (*Enterobacter cloacae, K. pneumoniae* and *E. coli* strains) have been present in Indian hospitals since 2006 and possibly even earlier [71]. NDM-producers have also been isolated from drinking and seepage water (i.e., pools of water in the streets) samples attained in New Delhi, which poses a major health threat to inhabitants relying on public sanitation facilities and tap water [72]. Since their discovery, NDM carbapenemases have been reported to be found in *Enterobacteriaceae* isolates worldwide, mostly from patients with travel history to India [73]. A study published in 2019 reported that NDM-producing *K. pneumoniae* isolates were isolated from hospitalized patients at a hospital in Tehran, Iran [74]. A study conducted in the United Arab Emirates, published in 2019, was concerned with CRE isolates carrying plasmids of the incompatibility group X type 3 (*IncX3*), these isolates were collected in the period between 2009 and 2014. Thirty isolates were found to harbor either *bla_NDM-1_, bla_NDM-4_, bla_NDM-5_, bla_NDM-7_, bla_OXA-181_* or *bla_KPC-2_* carbapenemase genes on *IncX3* plasmids. Phylogenetic analysis suggested that the detected carbapenemase genes did not evolve locally in the United Arab Emirates, but rather occurred due to international travel [75].

#### Oxacillinase

In 2001, OXA-48 was first identified in a *K. pneumoniae* isolate from Istanbul, Turkey [76]. Five years later, the first outbreak of infections caused by OXA-48-producing *K. pneumoniae* was reported in Istanbul [77]. In 2010, an outbreak caused by OXA-48-producing *K. pneumonia* isolates was reported in France [78]. Another outbreak was also reported in Belgium [79]. Hospital outbreaks have been reported in The Netherlands, as well as Russia [62]. Sporadic cases of OXA-48-producing isolates have been reported in Senegal [80], Lebanon [81], Tunisia [82] and Egypt [20].

A recent study conducted in Egypt on carbapenem-resistant Gram-negative bacteria recovered from febrile neutropenic pediatric cancer patients during the period from October 2014 to December 2016, revealed that *bla_OXA-48_* was the most prevalent carbapenemase gene (58.62%), followed by *bla_NDM_* (27.58%), *bla_VIM-3_* (10.3%) and *bla_KPC-2_* (6.89%) [83]. Another study conducted in Iran on carbapenem-resistant *K. pneumoniae* isolates from clinical samples of blood, urine and sputum, obtained from October 2015 to September 2016, revealed that *bla_OXA-48_* was the most prevalent carbapenemase gene (72%), followed by *bla_NDM_* (31%) [74]. A study published in 2018 on carbapenem-resistant *Acinetobacter baumannii* isolates from 14 Colombian hospitals detected *bla_OXA-23-_*-like and *bla_OXA-51_*_-_.like-genes occurring simultaneously in 97.5% of the tested isolates [84].

## Detection of CRE

Infections caused by CRE pose a major health threat since they are usually resistant to β-lactams, aminoglycosides and fluoroquinolones [85]. CRE are now causing treatment failure in both community-acquired and nosocomial infections [86]. Consequently, there is a serious need for rapid and accurate detection of carbapenemase-producing isolates. According to the CLSI guidelines, isolates of *Enterobacteriaceae* are suspected of being carbapenemase producers when the minimum inhibitory concentrations (MICs) of meropenem or imipenem are 2–4 μg/ml or MIC of ertapenem is 2 μg/ml [87].

### The modified Hodge test

Although this test is cheap and very simple to perform, a high frequency of false-positive results was observed with isolates that produce ESBLs associated with porin loss or alterations [88,89]. False negative results were also observed with NDM-1 carbapenemases [89]. For these reasons, this test was removed from CLSI guidelines in 2018.

### Carba NP test

The Carba NP test is a colorimetric microtube assay used to test for carbapenemase production in *Enterobacteriaceae* and *P. aeruginosa.* This test has a high level of sensitivity and specificity (>90% each) in detecting KPC, NDM, VIM, IMP and *S. marcescens* enzyme-type carbapenemases, but low sensitivity (11%) for detecting OXA-48 carbapenemases [87]. The Carba NP test was reported to detect carbapenemase production even in imipenem-susceptible carbapenemase-producing *Enterobacteriaceae* (CPE) [90]. A ready-to-use version (RAPIDEC^®^ Carba NP test) for routine use in laboratories has been recently made commercially available [91].

### Modified carbapenem inactivation method

The modified carbapenem inactivation method (mCIM) test is used to detect carbapenemase-production in *Enterobacteriaceae* and *P. aeruginosa.* Contrary to Carba NP, which requires special reagents that are not routinely used in clinical laboratories, the mCIM test uses readily available reagents and media. Its procedure is simple, and the results can be easily interpreted. Moreover, an EDTA-mCIM can be used along with mCIM to differentiate serine carbapenemases from MBLs in *Enterobacteriaceae* [87].

### Bioluminescence-based carbapenem susceptibility detection assay

This method was recently developed by Vincent van Almsick *et al*. It permits the identification of carbapenemase-producing *A. baumannii*, carbapenemase-producing-CRE and noncarbapenemase-producing-CRE in just 2.5 h from culture media with a sensitivity and specificity of 99 and 98%, respectively [92].

### Immunochromatographic assays

A number of immunochromatographic assays have been developed to enable the detection of VIM, NDM, KPC and OXA-48 carbapenemases in 5 min directly from cultured bacterial colonies [93]. These assays are based on monoclonal antibodies that were generated by immunization in mice [94].

### Matrix-assisted laser desorption-ionization time-of-flight mass spectrometry

In this method, freshly prepared bacterial cultures are mixed with carbapenem solutions such as ertapenem or meropenem and incubated for 2–4 h at 35–37°C. Afterwards the mixture is centrifuged and the mass spectrometry technique is used to measure the supernatant. In case of carbapenemase hydrolysis, the degradation products and sodium salt of the carbapenem molecule are visible in spectra [95,96].

### Spectrophotometric assay

This method is performed by preparing a bacterial crude extract, usually by sonication, which is then added to a buffered imipenem solution. UV spectroscopy is used to measure the hydrolysis of the β-lactam ring [96,97].

### Molecular assay

A number of molecular techniques for the detection of carbapenemase genes are currently available. These assays can determine not only the exact identity of the carbapenemase, but also the absence or presence of the enzyme(s) [89]. These assays include conventional simplex and multiple PCR assays, using the appropriate primers for each gene (Table 1). The hyplex SuperBug ID test system (bioTRADING, Mijdrecht, Netherlands) is one of the available PCR assays [98]. There are also loop-mediated isothermal amplification-based systems such as the eazyplex^®^ SuperBug CRE system (AmplexDiagnostics GmbH, Gars, Germany) [99]. Several real-time PCR assays are also available such as the NucliSENS EasyQ KPC assay (bioMérieux, Marcy-l'Étoile, France), the Check-Direct CPE assay (Check-points, Wageningen, The Netherlands) and the Xpert Carba-R assay (Cepheid Inc., CA, USA) [100–102].

**Table 1.** Primers sequences, PCR product sizes and annealing temperatures of carbapenemase genes.

Gene	Forward primer (5′ → 3′)	Reverse primer (5′ → 3′)	Expected product size (bp)	Ta (°C)	Ref.
*bla*_KPC_	TGTCACTGTATCGCCGTC	CTCAGTGCTCTACAGAAAACC	900	58	[63,103,104]
	CGTCTAGTTCTGCTGTCTTG	CTTGTCATCCTTGTTAGGCG	798	52	[28,105]
	CTGTCTTGTCTCTCATGGCC	CCTCGCTGTGCTTGTCATCC	796	53	[47]
*bla*_IMP_	CTACCGCAGCAGAGTCTTTG	AACCAGTTTTGCCTTACCAT	587	55	[104,106]
	GAAGGCGTTTATGTTCATAC	GTACGTTTCAAGAGTGATGC	587	60	[103]
	GGAATAGAGTGGCTTAAYTC^†^	TCGGTTTAAYAAAACAACCACC^†^	232	52	[28,105]
*bla*_VIM_	TCTACATGACCGCGTCTGTC	TGTGCTTTGACAACGTTCGC	748	50	[107]
	GTTTGGTCGCATATCGCAAC	AATGCGCAGCACCAGGATAG	389	60	[103]
	AGTGGTGAGTATCCGACAG	ATGAAAGTGCGTGGAGAC	261	52	[104,108]
	GATGGTGTTTGGTCGCATA	CGAATGCGCAGCACCAG	390	52	[28,105]
*bla*_NDM_	GGTTTGGCGATCTGGTTTTC	CGGAATGGCTCATCACGAT	621	50	[105,109]
	GCAGCTTGTCGGCCATGCGGGC	GGTCGCGAAGCTGAGCACCGCAT	782	60	[103]
	CAGCGCAGCTTGTCG	TCGCGAAGCTGAGCA	784	52	[110]
*bla*_OXA-48_	GCGTGGTTAAGGATGAACAC	CATCAAGTTCAACCCAACCG	438	52	[28,103,105]
	TTGGTGGCATCGATTATCGG	GAGCACTTCTTTTGTGATGGC	743	56	[111,112]

*bla*_KPC_. *bla*_IMP_. *bla*_VIM_. *bla*_NDM_ and *bla*_OXA-48_ genes code for KPC, IMP, VIM, NDM and OXA-48 carbapenemases, respectively.

† Y stands for C or T.

IMP: Imipenemase or imipenem-resistant Pseudomonas-type carbapenemases, class B; KPC: *Klebsiella pneumoniae* carbapenemases; NDM: New Delhi metallo-β-lactamase; Ta: Annealing temperature; VIM: Veronese imipenemase.

In a recent study conducted by Mentasti *et al*. in 2019, an assay targeting IMP, NDM, VIM, KPC and OXA-48-like carbapenemases was designed and validated for the rapid detection of the mentioned carbapenemases from Enterobacteriales and Gram-negative nonfermenter bacteria by real-time PCR and melt-curve analysis [113].

DNA microarray assays are currently available for use as well, including but not limited to the Check MDR CT103 XL kit (Check-points) [114].

Moreover, construction and evaluation of a microbiological positive process internal control for PCR-based examination of food samples for *Listeria monocytogenes* and *Salmonella enterica* was carried out where an assay for detecting a 76 bp fragment of the green fluorescent protein (GFP) from *Aequorea victoria* was carried out [115].

## Treatment options for infections caused by carbapenem-resistant bacteria

Glycopeptides are still considered as good alternatives to carbapenems in cases where the infection is caused by carbapenem-resistant Gram-positive bacteria. However, carbapenem-resistant Gram-negative bacteria, especially CRE, have limited treatment options since they usually carry resistance determinants to β-lactams, aminoglycosides and fluoroquinolones [116,117]. In such cases, treatment options should be discussed with microbiologists since some CREs are sensitive to amikacin. Older antimicrobials that were rarely administered in the past due to efficacy and toxicity concerns may be considered. These may include fosfomycin, polymyxins (colistin) and the newer tigecycline [85,118].

Dual-carbapenem combination therapy may be considered for infections caused by pandrug-resistant bacteria; however, data on this treatment are somewhat limited [119,120].

Some studies suggest *in vitro* synergistic effects of several antibiotic combinations against carbapenem-resistant Gram-negative bacteria. These synergistic combinations include colistin with rifampicin [121,122], carbapenem with sulbactam [122], colistin with carbapenem [123] and carbapenem with an aminoglycoside [124]. However, *in vivo* studies showed unexpected results. The colistin/meropenem combination *in vivo* did not result in better outcomes compared with colistin monotherapy in regard to either clinical response or development of resistance [125,126].

Plazomicin is a newly marketed next-generation aminoglycoside [31]. In a study conducted by Rodríguez-Avial *et al.*, Plazomicin was used at subinhibitory concentrations in combination with fosfomycin, meropenem and colistin. Results showed a synergistic bactericidal effect against carbapenemase-producing *K. pneumoniae* isolates [127]. Novel β-lactamase inhibitors, such as vaborbactam, avibactam and relebactam, are capable of counteracting the effect of KPC and ESBLs [128]. Lately, new β-lactam/β-lactamase inhibitor combinations, namely meropenem/vaborbactam, ceftazidime/avibactam and imipenem/cilastatin/relebactam were approved by the US FDA (MD, USA) for the treatment of infections caused by CRE [118,129,130].

A number of novel antimicrobial agents are being developed for the treatment of infections caused by resistant bacteria, cefiderocol (S-649266) is one of them. This siderophore cephalosporin reaches the periplasmic space by active transport and binds to PBP3 of Gram-negative bacteria, which eventually causes the inhibition of bacterial cell wall synthesis. Cefiderocol was reported to be stable to carbapenemases and other ESBLs [131–133]. Eravacycline is a new tetracycline with a broad spectrum of activity that includes CRE [134].

## Antimicrobial stewardship

Antimicrobial stewardship is a term referring to programs and coordinated interventions aiming at regulating the use of antimicrobials [135]. The main purpose of antimicrobial stewardship is to attain the best clinical outcomes regarding antimicrobial use, while reducing adverse effects and toxicity in order to limit the selective pressure on bacteria, which leads to the emergence of AMR [136]. Antimicrobial stewardship plans should be developed and implemented by all healthcare facilities following the Infectious Disease Society of America and Society for Healthcare Epidemiology of America (VA, USA) guidelines [137] and careful monitoring of these interventions is highly recommended [136].

## Conclusion

Carbapenems represent an important class of antibiotics that are still reserved for infections caused by MDR microorganisms. However, the emergence of carbapenem resistance has dramatically increased worldwide and therefore poses a serious public health threat. Several mechanisms including reduced uptake, active efflux of carbapenems, as well as inactivation via carbapenemases are involved in the bacterial resistance to carbapenems. A number of molecular assays for the detection of carbapenemase genes are currently available including conventional simplex PCR, multiplex PCR, real-time PCR and loop-mediated isothermal amplification-based systems. Glycopeptides, Fosfomycin, polymyxins (colistin), tigecycline, plazomicin and new members of tetracyclines such as eravacycline are the last treatment options for the treatment of infections caused by carbapenem-resistant bacteria. Therefore, the use of these last resort antibiotics should be controlled to avoid antibiotic misuse or overuse, and their use should be limited to the intensive care units in hospitals and only prescribed under strict medical supervision.

## Future perspective

The collection of epidemiological data is important for taking appropriate and yet affordable measures against CRE and CPE. Guidelines should be developed and implemented in all healthcare facilities to enable epidemiological data collection and rapid reporting of any outbreaks so that appropriate measures could be taken as soon as possible. Antibiotic stewardship programs should be implemented for antibiotic prescription and use, as well as for the control and monitoring of infections caused by the clinically relevant pathogens in healthcare facilities. Culture and sensitivity, as well as MIC determination, for carbapenem-resistant pathogens should be performed before initiating antimicrobial therapy to determine the appropriate dose and duration of treatment, and thereby avoid unnecessary prescription and overuse of carbapenems.

Continued research is urgently needed to determine the most appropriate treatment for serious CRE infections. Alternative approaches for treating such infections should be considered, such as phage therapy or quenching of quorum sensing. Antibiotic combinations that show promising *in vitro* effects should be investigated through clinical trials to determine their efficacy *in vivo.* As for antimicrobial development, guidelines should be implemented for premarketing research on potential mechanisms of resistance at an early stage of new antimicrobial development. Finally, strict measures must be taken to prevent dispensing of antimicrobials without a prescription to avoid the misuse or overuse of such agents.

**Executive summary**

- The emergence of carbapenem resistance has dramatically increased worldwide and resulted in treatment failure of community-acquired and nosocomial infections.
- Reduced uptake, active efflux and production of carbapenemases are the major resistance mechanisms to carbapenems.
- Conventional simplex, multiplex, real-time PCR and loop-mediated isothermal amplification–based systems are currently available techniques for the detection of carbapenemase genes.
- Glycopeptides, fosfomycin and polymyxins (colistin), plazomicin, tigecycline and new members of tetracyclines such as eravacycline are still effective for the treatment of infections caused by carbapenem-resistant pathogens.
